# Biochemical Recurrence and Risk of Mortality Following Radiotherapy or Radical Prostatectomy

**DOI:** 10.1001/jamanetworkopen.2023.32900

**Published:** 2023-09-11

**Authors:** Ugo Giovanni Falagario, Ahmad Abbadi, Sebastiaan Remmers, Lars Björnebo, Darko Bogdanovic, Alberto Martini, Alexander Valdman, Giuseppe Carrieri, Mani Menon, Olof Akre, Martin Eklund, Tobias Nordström, Henrik Grönberg, Anna Lantz, Peter Wiklund

**Affiliations:** 1 Department of Molecular Medicine and Surgery (Solna), Karolinska Institutet, Stockholm, Sweden; 2 Department of Urology and Kidney Transplantation, University of Foggia, Foggia, Italy; 3 Departments of Medical Epidemiology and Biostatistics, Karolinska Institutet, Stockholm, Sweden; 4 Department of Urology, Erasmus MC Cancer Institute, Rotterdam, the Netherlands; 5 Department of Urology, Division of Surgery, The Univeristy of Texas MD Anderson Cancer Center, Houston; 6 Department of Oncology-Pathology, Karolinska Institutet, Stockholm, Sweden; 7 Department of Urology, Icahn School of Medicine at Mount Sinai, New York, New York; 8 Department of Clinical Sciences, Danderyd Hospital, Karolinska Institutet, Stockholm, Sweden

## Abstract

**Importance:**

Stratifying patients with biochemical recurrence (BCR) after primary treatment for prostate cancer based on the risk of prostate cancer–specific mortality (PCSM) is essential for determining the need for further testing and treatments.

**Objective:**

To evaluate the association of BCR after radical prostatectomy or radiotherapy and its current risk stratification with PCSM.

**Design, Setting, and Participants:**

This population-based cohort study included a total of 16 311 male patients with 10 364 (64%) undergoing radical prostatectomy and 5947 (36%) undergoing radiotherapy with curative intent (cT1-3, cM0) and PSA follow-up in Stockholm, Sweden, between 2003 and 2019. Follow-up for all patients was until death, emigration, or end of the study (ie, December 31, 2018). Data were analyzed between September 2022 and March 2023.

**Main Outcomes and Measures:**

Primary outcomes of the study were the cumulative incidence of BCR and PCSM. Patients with BCR were stratified in low- and high-risk according to European Association of Urology (EAU) criteria.

**Exposures:**

Radical prostatectomy or radiotherapy.

**Results:**

A total of 16 311 patients were included. Median (IQR) age was 64 (59-68) years in the radical prostatectomy cohort (10 364 patients) and 69 (64-73) years in the radiotherapy cohort (5947 patients). Median (IQR) follow-up for survivors was 88 (55-138) months and 89 (53-134) months, respectively. Following radical prostatectomy, the 15-year cumulative incidences of BCR were 16% (95% CI, 15%-18%) for the 4024 patients in the low D’Amico risk group, 30% (95% CI, 27%-32%) for the 5239 patients in the intermediate D’Amico risk group, and 46% (95% CI, 42%-51%) for 1101 patients in the high D’Amico risk group. Following radiotherapy, the 15-year cumulative incidences of BCR were 18% (95% CI, 15%-21%) for the 1230 patients in the low-risk group, 24% (95% CI, 21%-26%) for the 2355 patients in the intermediate-risk group, and 36% (95% CI, 33%-39%) for the 2362 patients in the high-risk group. The 10-year cumulative incidences of PCSM after radical prostatectomy were 4% (95% CI, 2%-6%) for the 1101 patients who developed low-risk EAU-BCR and 9% (95% CI, 5%-13%) for 649 patients who developed high-risk EAU-BCR. After radiotherapy, the 10-year PCSM cumulative incidences were 24% (95% CI, 19%-29%) for the 591 patients in the low-risk EAU-BCR category and 46% (95% CI, 40%-51%) for the 600 patients in the high-risk EAU-BCR category.

**Conclusions and Relevance:**

These findings suggest the validity of EAU-BCR stratification system. However, while the risk of dying from prostate cancer in low-risk EAU-BCR after radical prostatectomy was very low, patients who developed low-risk EAU-BCR after radiotherapy had a nonnegligible risk of prostate cancer mortality. Improving risk stratification of patients with BCR is pivotal to guide salvage treatment decisions, reduce overtreatment, and limit the number of staging tests in the event of PSA elevations after primary treatment.

## Key Points

**Question:**

What is the association of biochemical recurrence (BCR) after primary treatment for prostate cancer and its current risk stratification with prostate cancer-specific mortality?

**Findings:**

In this large population-based cohort study of 16 311 male patients undergoing radical prostatectomy or radiotherapy, biochemical recurrence after primary treatment was found to be a common event with a limited association with cancer-related mortality. Risk groups at diagnosis, prostate specific antigen (PSA) velocity, and primary treatment were the most strongly associated with mortality factors.

**Meaning:**

These findings suggest that improving PSA recurrence definitions and risk stratification tools for patients with BCR may help to reduce overtreatment and overtesting.

## Teaser

This cohort study examines the association of biochemical recurrence with prostate cancer–specific mortality after radical prostatectomy or radiotherapy and its current risk stratification with prostate cancer mortality among patients in Sweden.

## Introduction

The most sensitive biomarker for prostate cancer recurrence after radical prostatectomy or radiotherapy is serum prostate specific antigen (PSA).^1^ However, the evidence on the association of biochemical recurrence (BCR) (ie, PSA rise after treatment with curative intent) with prostate cancer survival is limited. Patients with rising PSA after radical prostatectomy or primary radiotherapy have different risks of subsequent symptomatic metastatic disease based on various parameters, including the PSA levels. Moreover, the high variability in reported effect sizes of BCR suggests that only certain patient subgroups with BCR might be at an increased risk of mortality.^2^ In a recent systematic review of the literature, Van den Broeck et al^3^ found that higher International Society of Urological Pathology (ISUP) Gleason Grade Group (GG), shorter time from treatment to recurrence, and shorter PSA doubling time (PSA-DT) are associated with prostate cancer–specific mortality (PCSM) in patients with BCR. Based on these findings, the EAU guidelines suggest to stratify patients with BCR into EAU low-risk BCR (for radical prostatectomy: PSA-DT of more than 1 year and pathological GG of less than 4; for radiotherapy: time to BCR of more than 18 months and biopsy GG of less than 4) or EAU high-risk BCR (for radical prostatectomy: PSA-DT of less than 1 year or pathological GG 4 to 5; for radiotherapy: interval to biochemical failure of less than 18 months or biopsy GG 4 to 5).^2^ Subsequently, Tilki et al^4^ and Pak et al^5^ externally validated the proposed risk stratification for patients undergoing radical prostatectomy, suggesting that patients who are low risk may not require an immediate intervention at time of BCR.

The retrospective nature of most studies included in the systematic review by Van den Broeck et al,^3^ the heterogeneity in BCR definitions, number of PSA measurement, different calculations of PSA and PSA-DT, limit the quality of the evidence, and precluded further strong recommendations. By leveraging a large-scale, population-based Swedish database containing comprehensive information on treatment modalities, PSA testing, and survival outcomes, the primary objective of this study was to evaluate the association of BCR and its current risk stratification with PCSM.

## Methods

This cohort study was approved by the regional ethics board in Stockholm, Sweden, and informed consent was waived because deidentified retrospective data from a population-based registry was used. The study was reported following Strengthening the Reporting of Observational Studies in Epidemiology (STROBE) reporting guideline.

We used the Stockholm PSA and Biopsy Register, a population-based register that contains data on every PSA test and prostate biopsy taken in Stockholm County since 2003. We included all male patients who underwent radiotherapy or radical prostatectomy with curative intent (cT1-3, cM0) and at least 1 PSA after treatment (median, 10 [95% CI, 7-15] treatments). PSA measurements were performed in 3 centralized laboratories and were linked to the National Prostate Cancer Register (NPCR) of Sweden, the Prescribed Drug Register, the inpatient and outpatient registries, and the Swedish Cause of Death Register.^6^ Methods for assessment of cause of death in Swedish studies have been previously described.^7^

Follow-up for all patients was until death, emigration, or end of the study. The study end date was set to the last available update of the Swedish Death Register (December 31, 2018). BCR was defined as PSA of 0.2 or more in 2 consecutive measurements after radical prostatectomy, and PSA nadir of 2 ng/mL after radiotherapy. PSA-DT was computed using all PSA values until BCR with the Memorial Sloan Kettering Cancer Center method.^8^ A total of 587 patients with PSA persistence after radical prostatectomy were excluded. 

### Statistical Analysis

Primary outcomes of the study were the cumulative incidence of BCR and PCSM. Statistical analyses were performed in 2 steps and repeated for radical prostatectomy and radiotherapy group. First, we included all patients and set the date of treatment as time 0. We evaluated the cumulative incidence of BCR according to D’Amico risk groups at diagnosis. The competing risk was all-cause mortality, and patients undergoing any salvage treatment before recurrence were censored. Next, a competing-risks regression was fit to evaluate the association of having a low and high-risk BCR compared with not experiencing BCR on PCSM (with the competing risk of other cause mortality). The selection of covariates was guided by a combination of prior literature, clinical knowledge, and statistical considerations. The model ultimately included age at diagnosis, D’Amico risk groups, treatment year, Charlson comorbidity index (CCI), salvage treatment timing, and BCR risk groups. Salvage treatment timing and BCR risk groups, as per EAU, were treated as time-varying covariates.

Second, we included only patients who developed BCR (time 0, date of BCR), to validate EAU-BCR risk stratification and to evaluate factors associated with PCSM in this subset of patients. Two competing-risks regression models were built: 1 including EAU-BCR risk groups alone, and 1 including age, T stage, ISUP GG, time to BCR (continuous), and PSA-DT (continuous). Both the models were adjusted for salvage treatment (fitted as time-varying covariate) and concordance index (Harrell C index) was computed to evaluate discrimination of EAU-BCR risk groups alone vs a multivariable model in estimating PCSM. Statistical analyses were performed using Stata version 16 (StataCorp). Statistical analyses were performed between September 2022 and March 2023, and statistical significance was set at *P* < .05.

## Results

Overall, 16 311 patients were included. Reasons for patient exclusions are detailed in the eFigure in Supplement 1. Median (IQR) age was 64 (59-68) years in the radical prostatectomy cohort (10 364 patients) and 69 (64-73) years in the radiotherapy cohort (5947 patients). Median (IQR) follow-up for survivors was 88 (55-138) months and 89 (53-134) months, respectively. Clinical characteristics according to the treatment received are presented in Table 1. There were 8514 of 10 364 male patients (82%) who underwent radical prostatectomy with low comorbidities based on the CCI of 0 to 1 comorbidity while 1033 of 5947 (17%) showed low comorbidities in the radiotherapy cohort. Risk categories were skewed toward the lower end among male patients who underwent radical prostatectomy, whereas the risk categories were more evenly distributed among male patients who underwent radiotherapy.

**Table 1.** Demographic and Disease Characteristics of All Study Patients According to Initial Treatment Received

Characteristics	Patients, No. (%)	
	Radical prostatectomy (n = 10 364)	Radiotherapy (n = 5947)
Age, median (IQR), y	64 (59-68)	69 (64-73)
CCI		
0-1	8514 (82.1%)	1033 (17.4)
2	923 (8.9)	3861 (64.9)
≥3	927 (8.9)	1053 (17.7)
Biopsy ISUP GG		
1	4736 (45.7)	1806 (30.4)
2	3693 (35.6)	1796 (30.2)
3	1297 (12.5)	1184 (19.9)
4	420 (4.1)	599 (10.1)
5	218 (2.1)	562 (9.5)
PSA, median (IQR), ng/mL	5.9 (4.3-8.9)	8.5 (5.6-15.8)
PSA density, median (IQR), ng/mL/mL	0.2 (0.1-0.2)	0.2 (0.1-0.4)
D’Amico risk		
Low	4024 (38.8)	1230 (20.7)
Intermediate	5239 (50.5)	2355 (39.6)
High	1101 (10.6)	2362 (39.7)
cT stage		
cT1	6986 (67.4)	2780 (46.7)
cT2	3145 (30.3)	2147 (36.1)
cT3	233 (2.2)	1020 (17.2)
Adjuvant ADT		
No hormone	NA	1672 (28.1)
<6 mo	NA	901 (15.2)
6-12 mo	NA	1982 (33.3)
12-18 mo	NA	271 (4.6)
>18 mo	NA	1121 (18.8)
Salvage therapy		
No salvage treatment	8811 (85.0)	5122 (86.1)
ADT	166 (1.6)	825 (13.9)
RT	843 (8.1)	0
RT+ADT	544 (5.2)	0
Salvage therapy timing		
No salvage treatment	8811 (85.0)	5122 (86.1)
Before BCR	604 (5.8)	203 (3.4)
After BCR	949 (9.2)	622 (10.5)
Follow-up time for survivors, median (IQR), mo	88 (55-138)	89 (53-134)
Follow-up time from BCR for survivors who experienced BCR, median (IQR), mo	84 (51-135)	83 (46-127)

Abbreviations: ADT, androgen deprivation therapy; BCR, biochemical recurrence; cT, clinical T stage; CCI, Charlson comorbidity index; ISUP GG, International Society of Urological Pathology Gleason Grade Group; PSA, prostate specific antigen; RT, radiotherapy.

**Figure 1.**
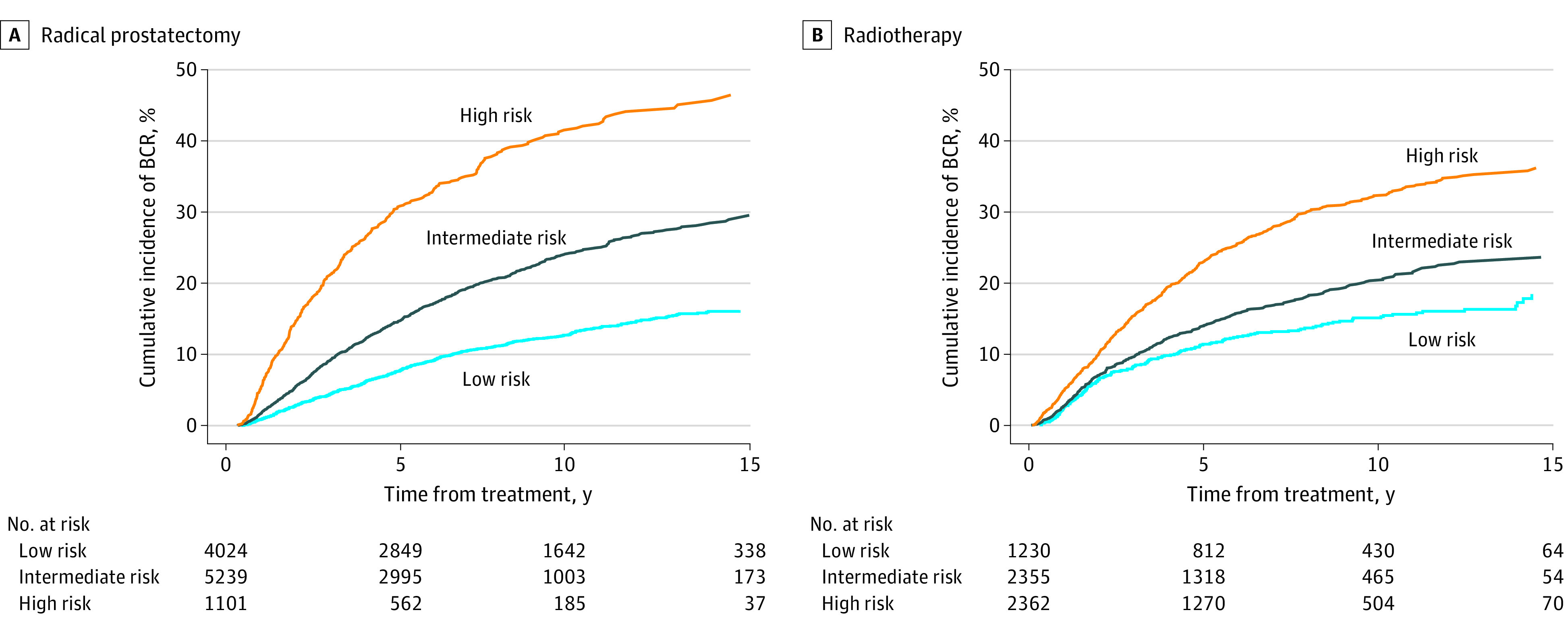
Cumulative Incidence Function Curves for Biochemical Recurrence (BCR) According to D’Amico Risk Groups at Diagnosis

**Figure 2.**
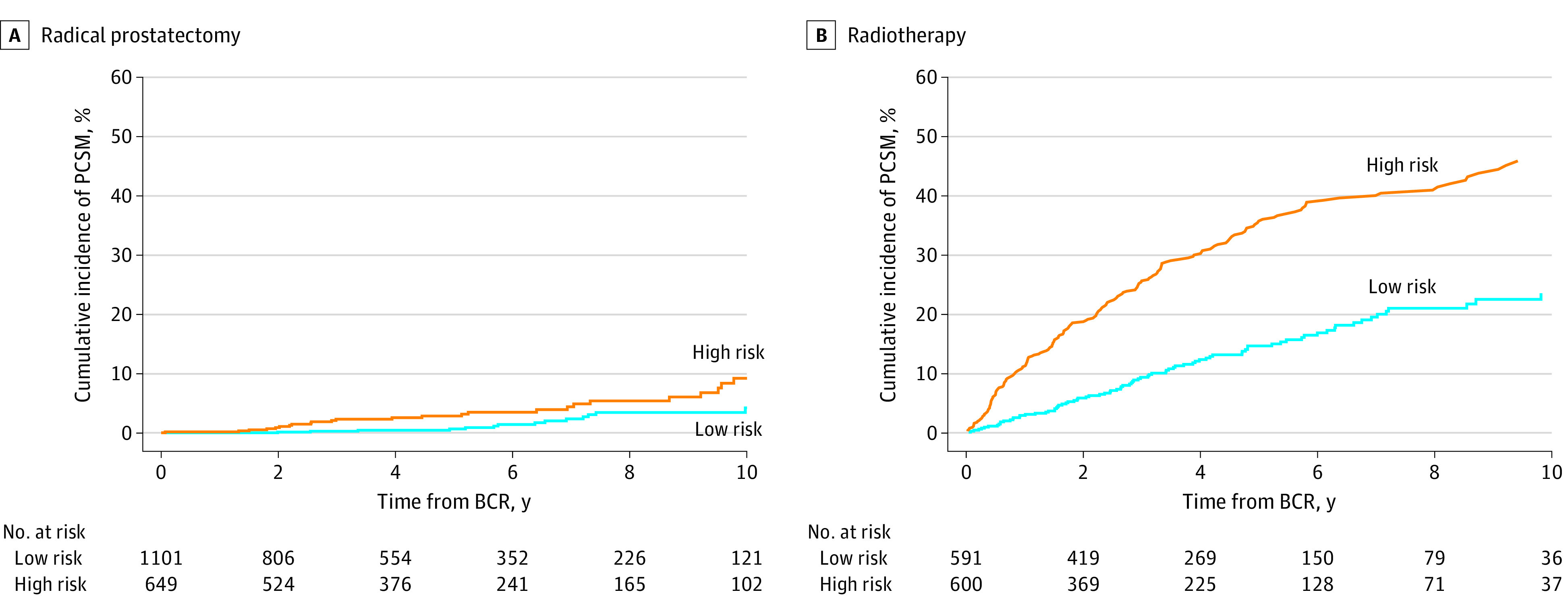
Cumulative Incidence Function Curves Demonstrating Prostate Cancer–Specific Mortality (PCSM) After Biochemical Recurrence (BCR) According to EAU BCR Risk Classification Adjusted for salvage treatment. Harrell C was 0.66 (95% CI, 0.57-0.75) in the radical prostatectomy group and 0.69 (95% CI, 0.66-0.72) in the radiotherapy groups.

Of the 10 364 male patients in the radical prostatectomy group, the cumulative incidences of BCR at 15 years after radical prostatectomy were 16% (95% CI, 15%-18%) for the 4024 male patients (39%) in the low D’Amico risk group, 30% (95% CI, 27%-32%) for the 5239 male patients (50%) in the intermediate D’Amico risk group, and 46% (95% CI, 42%-51%) for the 1101 male patients (11%) in the high D’Amico risk group (Figure 1). Compared with patients who did not experience BCR, falling in the EAU high-risk BCR category was a significant factor of PCSM (subdistribution hazard ratio [sHR], 1.27; 95% CI, 1.02-1.59), while having a low-risk BCR was not a significant factor of PCSM (sHR, 1.18; 95% CI, 0.96-1.46) (Table 2). The PCSM rates at 10 years after BCR adjusted for salvage treatment were 4% (95% CI, 2%-6%) and 9% (95% CI, 5%-13%) for patients who have low-risk and high-risk EAU-BCR, respectively (Figure 2).

**Table 2.** Competing-Risks Regression Analysis for Estimating Prostate Cancer Specific Mortality After Accounting for Other Cause Mortality in Overall Population

Covariate	Radical prostatectomy		Radiotherapy	
	sHR (95% CI)	*P* value	sHR (95% CI)	*P* value
Age at diagnosis, per 10 y	2.94 (2.07-4.20)	<.001	1.89 (1.61-2.23)	<.001
D’Amico risk				
Low risk	1 [Reference]	NA	1 [Reference]	NA
Intermediate risk	2.43 (1.14-5.16)	.02	1.13 (0.78-1.64)	.52
High risk	1.73 (0.68-4.44)	.25	1.22 (0.84-1.76)	.30
Treatment year, per year	0.93 (0.86-1.01)	.09	0.95 (0.92-0.97)	<.001
CCI, per unit	2.68 (1.49-4.79)	.001	5.58 (3.71-8.40)	<.001
Adjuvant ADT				
None	NA	NA	1 [Reference]	
Yes	NA	NA	1.88 (1.36-2.61)	<.001
Salvage therapy timing^a^				
None	1 [Reference]	NA	1 [Reference]	NA
Before BCR	1.14 (0.91-1.43)	.27	1.08 (1.00-1.17)	.05
After BCR	1.25 (1.00-1.56)	.05	0.99 (0.95-1.04)	.82
BCR^a^				
No BCR	1 [Reference]		1 [Reference]	
Low-risk EAU BCR	1.18 (0.96-1.46)	.12	1.34 (1.22-1.47)	<.001
High-risk EAU BCR	1.27 (1.02-1.59)	.03	1.45 (1.32-1.60)	<.001

Abbreviations: ADT, androgen deprivation therapy; BCR, biochemical recurrence; CCI, Charlson comorbidity index; EAU, European Association of Uology; NA, not applicable; sHR, subdistribution hazard ratio.

^a^ Included as time-varying covariate.

For the 5947 patients in the radiotherapy group, the cumulative incidences of BCR at 15 years after radiotherapy were 18% (95% CI, 15%-21%) for the 1230 male patients (21%) in the low D’Amico risk group, 24% (95% CI, 21%-26%) for the 2355 male patients (39%) in the intermediate D’Amico risk group, and 36% (95% CI, 33%-39%) for the 2362 male patients (40%) in the high D’Amico risk group (Figure 1). On multivariable competing risks regression, compared with not experiencing BCR, both low-risk BCR (sHR, 1.34; 95% CI, 1.22-1.47) and high-risk BCR (sHR, 1.45, 95% CI, 1.32-1.60) emerged as significant factors of PCSM (Table 2). Cumulative incidences of PCSM 10 years after BCR adjusted for salvage treatment were 24% (95% CI,19-29) for patients with low-risk EAU-BCR and 46% (95% CI, 40%-51%) for patients with high-risk EAU-BCR (Figure 2).

The discriminatory ability of the models including solely EAU-BCR risk groups adjusted for salvage treatment was 0.66 (95% CI, 0.57-0.75) in the radical prostatectomy group and 0.69 (95% CI, 0.66-0.72) in the radiotherapy group. The multivariable models including clinical variables had higher C index (radical prostatectomy: 0.72; 95% CI, 0.61-0.83; radiotherapy: 0.72; 95% CI, 0.69-0.76) and showed that PSA-DT was the strongest factor of PCSM after radical prostatectomy (eTable in Supplement 1).

## Discussion

To the best of our knowledge, this is the first population-based study that evaluated BCR risk stratification after both radical prostatectomy and radiotherapy. In addition, we estimated the association of having a high-risk BCR or low-risk BCR during follow-up after treatment with curative intent on prostate cancer-specific survival. We found that patients falling in the low-risk BCR category after radical prostatectomy had a similar risk of PCSM compared with patients with no BCR and may be considered as patients with nonclinically significant recurrence. Notably, the cumulative incidence of BCR was higher after radical prostatectomy than radiotherapy. However, the risk of dying from prostate cancer after BCR was higher after radiotherapy. We acknowledge that radical prostatectomy was more commonly performed in younger, healthier patients, while radiotherapy is often favored for patients who are older and more sick. This differential selection introduces the possibility of confounding by indication, where underlying patient characteristics associated with treatment choice may influence the observed outcomes.^9^ By including D’Amico risk groups, age, and comorbidities in the competing risk regression, we attempted to provide a more accurate assessment of the association between BCR and PCSM within each study group. Nevertheless, it is worth noting that the disproportion between the incidence of recurrence (higher in the radical prostatectomy group despite the lower risk profile) and mortality (higher in the radiotherapy group despite the lower risk of recurrence) may be further accentuated when comparing patients with similar risk profiles.

These results must be viewed considering the importance of BCR definitions in clinical practice.^10^ BCR is the earliest sign of disease recurrence and usually prompts additional testing and treatment.^11^ Additionally, its occurrence has a strong psychological impact on patients. On the other side, the Prostate Testing for Cancer and Treatment trial showed a low probability of PCSM at 15 years regardless of the initial treatment modality. Limiting salvage treatments to patients who might benefit from them should be considered a priority to avoid overtreatment.^12^ Development of prediction models, including PSA-DT and time to recurrence as continuous variables, may improve our clinical decision-making in this subset of patients.^13,14^

### Strengths and Limitations

This study’s strengths included the population-based design and the standardization of the acquisition of PSA values centralized to only 3 laboratories. However, this study had limitations. It was primarily limited by its retrospective design. Several modifications in the diagnostic and treatment pathway of patients at risk of prostate cancer have occurred in recent years, including the introduction of magnetic resonance imaging and target biopsies in the diagnostic algorithm, increasing use of active surveillance,^15,16^ and updates in the Gleason scoring system.^17^ Additionally, Swedish guidelines recommendations on the use and duration of ADT in patients undergoing radiotherapy have changed over the years. As previously reported, more than 90% of patients with high-risk prostate cancer underwent combined treatment with ADT and radiotherapy. In contrast, the use of ADT decreased in male patients with low-risk prostate cancer (from 31% in 2006 to 16% in 2012) and intermediate-risk prostate cancer (from 62% in 2006 to 43% in 2012).^18^ Additionally, the lack of information on metastatic status hinders a comprehensive evaluation. The diagnosis of metastases depends on the timing and imaging modality used and is not reliable in a retrospective setting.^19,20,21^

## Conclusions

In this cohort study, we validated EAU-BCR risk grouping and confirmed that male patients with low-risk BCR after radical prostatectomy might consider undergoing immediate salvage treatment. However, we found that while the probability of BCR was significantly higher after radical prostatectomy, the risk of dying from prostate cancer was higher after radiotherapy. This difference between the number of patients with defined disease recurrence and the risk for PCSM highlights 3 main concepts. First, BCR, as currently defined, is not a reliable estimator for PCSM and should not be used to compare treatment modalities. Second, patients with low-risk BCR, as per EAU definition, after radiotherapy have a relatively high risk of prostate cancer death, indicating the need for more stringent criteria. Third, risk stratification of patients with BCR is pivotal to guide salvage treatment decisions, reduce overtreatment, and limit the number of staging tests in the event of PSA elevations after primary treatment.

## Notes

Supplement 1. **eFigure.** Study Flowchart With Inclusion and Exclusion Criteria **eTable. **Competing-Risks Regression Analysis for Predicting Prostate Cancer Specific Mortality, After Accounting for Other Cause Mortality in Patients Who Developed Biochemical Recurrence (BCR) After Active Treatment

Supplement 2.
Data Sharing Statement
