# Supplementary material for: Biochemical Recurrence and Risk of Mortality Following Radiotherapy or Radical Prostatectomy
Source: JAMA Netw Open. 2023 Sep 11;6(9):e2332900. doi: 10.1001/jamanetworkopen.2023.32900 (PMC10495864; doi:10.1001/jamanetworkopen.2023.32900)
Supplement: Supplement 1. — eFigure. Study Flowchart With Inclusion and Exclusion Criteria eTable. Competing-Risks Regression Analysis for Predicting Prostate Cancer Specific Mortality, After Accounting for Other Cause Mortality in Patients Who Developed Biochemical Recurrence (BCR) After Active Treatment [file jamanetwopen-e2332900-s001.pdf]

Supplemental Online Content

Falagario UG, Abbadi A, Remmers S, et al. Biochemical recurrence and risk of mortality following radiotherapy or radical prostatectomy. *JAMA Netw Open*. 2023;6(9):e2332900. doi:10.1001/jamanetworkopen.2023.32900

eFigure. Study Flow Chart With Inclusion and Exclusion Criteria
eTable. Competing-Risks Regression Analysis for Predicting Prostate Cancer Specific Mortality, After Accounting for Other Cause Mortality

This supplemental material has been provided by the authors to give readers additional information about their work.

© 2023 Falagario UG et al. *JAMA Network Open.*

eTable 1. Competing-risks regression analysis for predicting prostate cancer specific mortality, after accounting for other cause mortality in patients who developed biochemical recurrence (BCR) after active treatment.

	**Radical Prostatectomy** Harrell's c 0.72 (0.61, 0.83)			**Radiotherapy** Harrell's c 0.72 (0.69, 0.76)		
**Covariate**	**SHR**	**95% CI**	**P>|z|**	**SHR**	**95% CI**	**P>|z|**
**Age at diagnosis, per 10ys**	3.65	2.48, 5.37	**<0.001**	1.30	1.1, 1.53	**0.002**
**T Stage***						
≤T2	Ref.			Ref.		
≥T3	1.11	0.6, 2.06	0.745	1.51	1.17, 1.95	**0.002**
**ISUP GG***						
≤3	Ref.			Ref.		
4-5	0.95	0.45, 1.99	0.883	1.66	1.29, 2.13	**<0.001**
**Time to BCR, per y**	1.04	0.86, 1.25	0.690	0.71	0.65, 0.79	**<0.001**
**PSA DT, per y**	0.97	0.95, 1	**0.019**	1.00	0.99, 1.01	0.992
**Salvage treatments§**						
None	Ref.			Ref.		
Yes	2.26	1.04, 4.92	**0.041**	1.05	0.98, 1.12	0.195

ISUP GG: International Society of Urological Pathology Gleason Group
* Clinical T stage and Biopsy Gleason Grade for RT patients, RP T stage and RP Gleason Grade for RP patients.
§ Included as Time Varying covariates

© 2023 Falagario UG et al. *JAMA Network Open.*

eFigure 1. Study flow chart with inclusion and exclusion criteria.

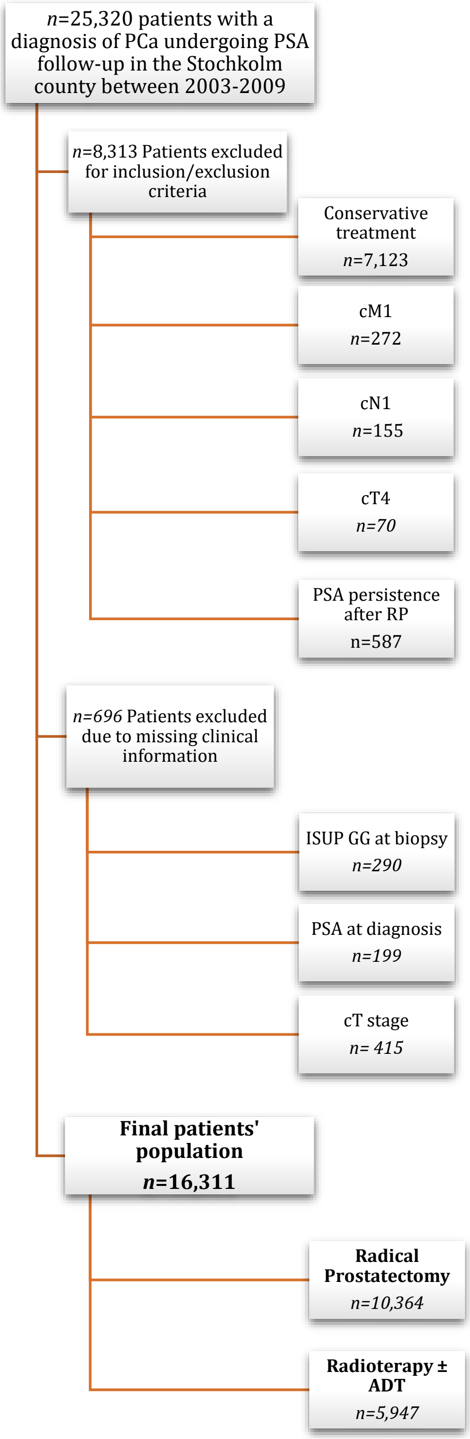

© 2023 Falagario UG et al. *JAMA Network Open.*
