# Supplementary material for: Biochemical Recurrence and Risk of Mortality Following Radiotherapy or Radical Prostatectomy
Source: JAMA Netw Open. 2023 Sep 11;6(9):e2332900. doi: 10.1001/jamanetworkopen.2023.32900 (PMC10495864; doi:10.1001/jamanetworkopen.2023.32900)
Supplement: Supplement 2. — Data Sharing Statement [file jamanetwopen-e2332900-s002.pdf]

Data Sharing Statement

Falagario. Biochemical Recurrence and Risk of Mortality Following Radiotherapy or Radical Prostatectomy. *JAMA Netw Open*. Published September 08, 2023.
doi:10.1001/jamanetworkopen.2023.32900

Data
Data available: No
